# 

**Published:** 1849

**Authors:** 


### ARTICLE V.

### TO OUR PATRONS.

As this number closes the volume, we deem it a proper time to make a few very brief requests of our subscibers, which we hope they will deem reasonable, and comply with.

1. We desire that all arrearge be settled by prompt remittance, and those who continue to take the work, will please remember that our terms are payment *in advance*.

2. We desire that all those who wish the work discontinued will notify us through the Post Master before the issue of the first number of the next volume which will be about a week before the first of May. This will be better than refusing a number as it saves us the postage and prevents the number from being spoiled.

3. We hope that no more subscribers will be so unjust as to take the first numbers and afterwards refuse them, as it gives us broken setts of the work, and does not relieve them of their liability for the whole volume.

4. We request all to remember, that letters sent without payment of postage are liable to not reach us at all.

5. And further we request our friends to aid in extending our circulation. We shall print twelve hundred and fifty copies, which will give us enough to supply some two hundred additional subscribers.

We have a number of extra copies of the current volume, which we will furnish to new subscribers, who send us three dollars in advance, with the next year's subscription to the work. As there will be a slight variation in the terms, the reader is referred to the Prospectus of next volume on the cover.

### INDEX.

Abdominal Movements, by Dr. McGirr, 203-287
Address to the Graduates of Rush Medical College, by Dr. Brainard, 480
Adipose Tumors, Plummer on, 206
Adulterated Drugs, Importation of, 184
American Medical Association 180
" " " Transactions of, 417
Amputation, Statistics of, in New York Hospital, 430
Amygdalitis, Chronic, 242
Ancient Monuments of Mississippi Valley, 447
Annual Commencement of Rush Medical College 547
Anti Mercury and Anti Mineral Doctors 334
Anthropotoxicologia 339
Aphonia and Stammering, by Dr. Lee 436
Asclepias Tuberosa 166
Association of Superintendents of Instiution for the Insane 263
Autopsy of a case of Absess of the Liver, by H. Rosenkrans M. D. 381
Baron Berzelius 352
Bartlett on Certainty in Medicine, Notice of 408
Beck on Infant Therapeutics, Notice of 498
Bedsores, mode of preventing 176
Bell and Stokes Practice. Notice of 415-611
Belladonna as an application to Ulcers, by E. S Cooper M. D. 471
Blake, Interesting cases, by 158
Bowers Memoranda of Anatomy, Surgery and Physiology 419
Brackett on Remedy for Deafness 467
Brain, Solly on Notice of 143-211
Brainard on Traetment of Spina Bifida, by Injections of Iodin, 495
Brainards Case of ununited faction of the Femur 187
Cataract Medical Treatment of 273
Cerebal Abscess, by Dr. Hall 291
Cerebral Circulaton, Notice of Burrows on 228
Chloride of Zinc, poisoning by 521
Chloroform, influence of on the blood 177
" " " Its dangers 527
" " " In Obstetrics, Eve on 70
" " " Simpson & Meigs on 151
Cholera Infantum, Observations on, by Dr. McNutt, 116
Cholera, 356, 357, 452
in New Orleans, 519
Electrical Phenomena in, 449
Report on, by committee of Board of Health of New York, notice of, 498
Treatment of, 420
Russian Vapor Bath in, 527
Christison's Dispensator, noticeof, 413
Coffee a disinfecting agent, 260
Consumption, Alcohol in, 361
Convention, Western Medical, 91
" of West. Medical Schools, 366, 453, 532
Cooper on Belladonna as an application to ulcers, 471
Copeland on Fever. 253
Creosote in Erysipelas, 449
Croup, Anodyne Treatment of, 350
Curiositios of Medical Science, 345
Deafness, Remedy for, by Dr. Brackett, 467
Diseases of the West—their complications, by Dr. Matthews, 382
Dislocation, old, Huber on, 104
Druitt's Surgery, notice of. 416
Dufton on the Ear, notice of, 399
Dunglison's Medical Dictionary notice of, 417
Dunlap on treatment of Hydrocele, 110
Edwards', Dr. W. G., letter from Paris, 393
Editorial changes, 540
Electrical phenomena in Cholera, 449
Ergot, Sanders' Strictures on the use of, 195
Ether and Chloroform, effects of, on animal temperature, 178
Ethics, Medical, 57
Euonymus Atropurpureus. 73
Evans on Retroveriion and Anteversion of the Uterus, 17
Excerpta. 275
Females and tneir Diseases, by Dr. Meigs, notice of. 54
Fitch on Medical Literature, 102
Foreign Bodies in the Stomach, 252
Garrod on Scurvey, 255
Geneva Medical College, notice of Circular of, 512
Gooch's Diseases of Women, notice of, 418
Graduates of Rush Medical College, 185
Graves and Gerhard's Clinical Medicine, notice of, 512
Greggs case of Death from Vaccination 109
Graduates of Rush Med Col., 547
Hall's case of Cerebral Abscess, 291
Hall, Letter from Dr., 531
Hamill's case of Polypus Uteri, 390
Harrison's Anatomy, notice of, 509
Headache, certain forms of, 324
Heart, Malformation of, by J. Jackson, M. D., 469
Hæmaturia, Flint's case of, 332
Hemorrhage, Ol. Turpentine in, 360
Hernia Cerebri, by Dr. Mears, 282
Herrick's case of Tracheotomy, 387
Holmes' Introductory Lecture, notice of. 133
Hood's Illustration of Hydropathy, 530
Horsley's case of Strangulated Hernia mistaken for Cholic—Consequent Death—by a Thomsonian, 386
Hospital for Insane of Indiana, notice of Report of, 501
Hospital for Insane of Illinois, notice of Report of, 503
Huber on old Dislocation of Humerus, 104
Huber on Placenta Praevia, 296
Human Combustion, Spontaneous, 250
Hydrocele, treatment of, by Dr. Dunlap, 110
Hydrocephalus, Iodide of Petassa in, 271
Imposition in the sale of Medicines, 5[illegible]3
Interesting cases, by Prof. Blake, 158
Intermittent Fevers, 446

Inflammation of the Faucis and Glottis —Obstruction—Tracheotomy, by J. B. Herrick, M. D., 387
Intermittent Diseases, pathology of, 395
Intestines, case of Abnormal position of, 114
Introductory Lectures, notice of, 504
Insane, Chicago Retreat for, 56
Iodine an Antidote to the venom of the Rattle Snake, by Dr. Whitmire, 396
Iron Rod, passage of, through the Head, 513
Irwin's case of Spinal Disease, 477
Jackson on Malformation of the Heart, 469
Jackson on Influence of the functions of the Skin in producing Fever, 475
Journal, North Western Medical and Surgical, 90
Kosciusko County Medical Society, 454
Labor, severe pain following, 273
Letter from Paris, by W. G. Edwards, M. D. 393
Life, Restoration to 164
Materia Medica, by Paine, notice of, 231
Malignant Pneumonia, Dr. Parks on, 461
Mal-Practice, Trial for, 586
Mammary Abscess, treatment of, 258
Matteucci's Lectures, notice of, 232
Matthews on Intestinal Worms, 11
" on Diseases of the West, &c., 382
Mears on Hernia Cerebri, 282
McGirr on Abdominal Movements, 203-287
McNutt on Cholera Infantum. 116
Medical Department of University of Michigan, 533
Medical Ethics, 85
Med. Intelligence, 92-186-275-366-460-549
Medical Legislation, 86
Medical Literature, Fitch on, 102
Medical Societies, 121
Medical Society of Northern Indiana, 269
Medical Topography of Greencastle, Indiana, and its vicinity, by Doctor Curran, 277
Meek on Purulent Ophthalmia, 95
Meek's Review of Meig's on Children, 300
Midwifery, Churchill's notice of, 144
Monster with a Head like a Dog's, 455
Mothershead on Sanguinaria Canadensis, 369
Natural Philosophy by Bird, notice of, 231
Naval Medical Corps, 346
Neuralgia, Osborne's case of, 298
New Medical Doctrine, 179
New Medical Schools, 354, 364
Obituary 276-550
Obstetrical Remembrancer, notice of, 146
Old Dislocation, case of, 104-193
Ophthalmia, Meek on, 95
Osborne's case of Neuralgia, 298
Ottawa Medical Society, 535
Parks on Puerperal Fever complicated with Malaria, 374
Parks on Malignant Pneumonia, 461
Pathology, Stille's, notice of, 25
Paine's Materia Medica, notice of 231
Peoria District Medical Society, 183
Phthisis Pulmonalis, by Dr. Bennet, 167
Physiology, Todd and Bowman's, notice of, 211
Placenta Prævia, new treatment of, Dr. Huber on, 296
Poisoning by Chloride of Zinc, 521
Poisoning by Coculus Indicus, Rosenkrans on, 295
Polypus Uteri, Dr. Hamill on, 390
Plummer on Adipose Tumors, 206
Puerperal fever complicated with malaria, Dr. Parks on, 374
Purpurea Hemorrhagica, Wadsworth on 107
Quackery, influence of statistics on, 80
Quinine, influence of, on the spleen, 428
Reese's Medical Lexicon, notice of, 510
Rheumatic affections of the stomach, by R. R. Stone, M. D.. 379
Rosenkrans' Autopsy of case of hepatic abscess, 381
Rosenkrans' case of poisoning, 295
" case of old dislocation, 193
Rush Medical College, 274-548
" " " annual commencement of, 547
Sanders' Strictures on the use of Ergot, 195
Sanguinaria Canadensis, Mothershead on, 369
Sargent's Minor Surgery, notice of, 322
Scurvy, Remarks on, 76
Sectional Medicine, 457
Semieology of the Tongue, 426
Skin, Jackson on influence of disordered functions of, in producing fevers, 475
Spasmo-paralysis by Marshal Hall, 160
Spermatic discharges, 244
Spermatorrhœa, Lallemand on, notice of, 126
Spina Bifidia, Brainard on, 495
Spinal Disease, Irwin's case of 477
Stahls Translation of Waller on Pylephlebitis, 1
Stahl's Translation of a case of malposition of the intestines, 114
Stille's Pathology, notice of, 25
Stomach, can a reptile live in the, 447
Stone on rheumatic affections of the stomach, 379
Stone's case of uterine mole, 208
Strangury, Caustic Potash in, 358
Strangulated hernia mistaken for cholic, Horsley's case of, 386
Surgery, McClellan's, notice of, 85, 145
Tears, Diagnostic value of, 251
To our patrons. 534
Tracheotomy, Herrick's case of, 387
Trial of a physician, 261
Twins born after an interval of two months, 252
Unavoidable Hemorrhage, 248
Ununited fracture, Brainard's case of, 187
Uterine mole, Stone's case of, 208
Uterus, Evans on Retroversion etc., of, 17
Vaccination, death following, Dr. Gregg's case of. 109
Vagina, stricture of, 259
Varicocele, new operation for, 450
Vena Portæ, inflammation of the, 1
Wadsworth's case of purpurea hemorrhagica, 107
Whitehead on abortion and sterility, 415
Whitmire on iodine as an antidote to the venom of the rattle snake, 396
Wilson's Anatomy, notice of, 322
Worms in the adult, Matthews on, 11
Wounds, new method of uniting, 147
Young stethoscopist, notice of, 144

### RECEIPTS FOR THIS JOURNAL FOR JANUARY AND FEBRUARY, 1849.

**INDIANA.**

| | |
|---|---|
| Drs. J. Collings, | $2 00 |
| T: W: Meller, | 2 00 |
| R. K. Long, | 1 00 |
| G. C. Paramore, | 2 00 |
| F. O. Miller, | 2 00 |
| G. N. Fitch, | 2 00 |
| W. H. Gifford, | 2 00 |
| R Lee, | 2 00 |
| W: E Sarber, | 2 00 |
| J. Rea, | 2 00 |
| H. D. Taylor, | 1 00 |
| Shively & Marshall, | 2 00 |
| Harris & Godwin, | 2 00 |

**ILLINOIS.**

| | |
|---|---|
| [illegible]rs. C. [illegible]. Richings, | $2 00 |
| G[illegible]. Southwich, | 2 00 |
| [illegible] Goodwin, | 2 00 |
| [illegible] | 2 00 |
| C: A: Hathwell, | 2 00 |
| E. G. Mygatt, | 2 00 |
| J. Kable, | 2 00 |
| J. W. Spaulding, | 2 00 |
| N. P. Holden, | 2 00 |
| D. B. Jewell, | 2 00 |
| A. E. Shepherd, | 6 00 |

**WISCONSIN,**

| | |
|---|---|
| Drs. S. Blood, | $2 00 |
| R. Hull, | 2 00 |
| J. H Turner, | 00 |
| T. G. Cole, | 1 00 |
| J. C. Miles, | 1 00 |

**MICHIGAN.**

| | |
|---|---|
| Drs. J. S. Skinner | $2 00 |
| W: B: Lincoln, | [illegible] 00 |
| L. W. Lovell | 2 00 |
| Campbell & Cox, | 2 [illegible]0 |

**OHIO.**

### NOTICE TO READERS AND CORRESPONDENTS.

We have received original communications from Drs. Howland, Thompson Mead, and Hathwell, which, with others already noticed will appear in our next number.

We have received, besides those noticed in the body of this number, the following pamphlets:

Dr. Mauley's Anniversary Discourse delivered before the New York Academy of Medicine.

The plea of Humanity in behalf of Medical Education. The Annual Address delivered before the New York State Medical Society. By A. H. Stevens, M. D., &c.

The Sixth Annual Report of the Managers of the State Lunatic Asylum, Utica, New York.

The Second Annual Report of the Trustees of the Indiana Institute for the Blind.

The Transactions of the College of Physicians and Surgeons of Philadelphia, up to January 2d, 1849.

Report of the Pennsylvania Hospital for the Insane, for the year 1848.

Transactions of the Medical Society of the State of New York for 1848.

Dr. Carnochan's pamphlet on congenital dislocation of the head of the femur on the dorsum of the ilium.

Report of the Eastern Asylum, in the city of Williamsburgh, Virginia.

Barrington and Haswell's Medical Bulletin.

---

### RUSH MEDICAL COLLEGE.

PROSPECTUS OF THE SECOND VOLUME OF THE

### NORTH-WESTERN MEDICAL AND SURGICAL JOURNAL.

EDITED BY

W. B. HERRICK, M. D., AND JOHN EVANS, M. D.

PROFESSORS IN RUSH MEDICAL COLLEGE.

This work, as heretofore, will be issued simultaneously at Chicago, Illinois, and Indianapolis, Indiana, on the first of every other month, beginning with May, in numbers, each containing from 88 to 96 pages, making, at the end of the year, a volume of about 550 pages, octavo, with title page and index complete.

It will be edited by W. B. HERRICK, M. D., and JOHN EVANS, M. D., Professors in Rush Medical College, Chicago, Illinois.

As the list of contributors to the work is annually increasing, it may be expected to furnish as good a variety of interesting original matter as any work of the kind in the country. The diseases of the west will claim especial attention.

Contributions to our pages, especially from physicians of the North-west, are earnestly solicited.

TERM .—Two DOLLARS per annum, *in advance*, which may be paid to any of our authorized agents, or sent to us by mail at our risk, directed to the "North-Western Medical and Surgical Journal, Chicago, Illinois." If payment be delayed until the close of the volume, three dollars will be invariably charged. No subscription received for less than an entire volume.

A list of receipts will be published in each number.

CHICAGO:

PRINTED BY J. W. DUZAN,

*Corner of Clark and Randolph Streets.*

